# Supplementary material for: Smooth Interpolating Curves with Local Control and Monotone Alternating Curvature
Source: Comput Graph Forum. 2022 Oct 6;41(5):25–38. doi: 10.1111/cgf.14600 (PMC9827861; doi:10.1111/cgf.14600)
Supplement: Supplementary file 1 — Supplement Material [file CGF-41-25-s001.zip › Local-Smooth-Interpolating-MonoCurvature/extern/clothoids/submodules/Utils/ThirdParties/fmt/fmt-8.0.1.zip!/fmt-8.0.1/doc/html/usage.html]

Usage — fmt 8.0.1 documentation


Toggle navigation

{fmt}

- 8.0.1
  - 8.0.1
  - 8.0.0
  - 7.1.3
- Contents
- Usage
  (current)
- API
- Syntax

# Usage¶

To use the {fmt} library, add `fmt/core.h`, `fmt/format.h`,
`fmt/format-inl.h`, `src/format.cc` and optionally other headers
from a release archive or
the Git repository to your project.
Alternatively, you can build the library with CMake.

## Building the Library¶

The included CMake build script can be used to build the fmt
library on a wide range of platforms. CMake is freely available for
download from https://www.cmake.org/download/.

CMake works by generating native makefiles or project files that can
be used in the compiler environment of your choice. The typical
workflow starts with:

```
mkdir build          # Create a directory to hold the build output.
cd build
cmake ..  # Generate native build scripts.
```

where `<path/to/fmt>` is a path to the `fmt` repository.

If you are on a \*nix system, you should now see a Makefile in the
current directory. Now you can build the library by running **make**.

Once the library has been built you can invoke **make test** to run
the tests.

You can control generation of the make `test` target with the `FMT_TEST`
CMake option. This can be useful if you include fmt as a subdirectory in
your project but don’t want to add fmt’s tests to your `test` target.

If you use Windows and have Visual Studio installed, a `FMT.sln`
file and several `.vcproj` files will be created. You can then build them
using Visual Studio or msbuild.

On Mac OS X with Xcode installed, an `.xcodeproj` file will be generated.

To build a shared library set the `BUILD_SHARED_LIBS` CMake variable to
`TRUE`:

```
cmake -DBUILD_SHARED_LIBS=TRUE ...
```

To build a `static library` with position independent code (required if the main
consumer of the fmt library is a shared library i.e. a Python extension) set the
`CMAKE_POSITION_INDEPENDENT_CODE` CMake variable to `TRUE`:

```
cmake -DCMAKE_POSITION_INDEPENDENT_CODE=TRUE ...
```

## Installing the Library¶

After building the library you can install it on a Unix-like system by running
**sudo make install**.

## Usage with CMake¶

You can add the `fmt` library directory into your project and include it in
your `CMakeLists.txt` file:

```
add_subdirectory(fmt)
```

or

```
add_subdirectory(fmt EXCLUDE_FROM_ALL)
```

to exclude it from `make`, `make all`, or `cmake --build .`.

You can detect and use an installed version of {fmt} as follows:

```
find_package(fmt)
target_link_libraries(<your-target> fmt::fmt)
```

Setting up your target to use a header-only version of `fmt` is equally easy:

```
target_link_libraries(<your-target> PRIVATE fmt::fmt-header-only)
```

## Usage with build2¶

You can use build2, a dependency manager and a
build-system combined, to use `fmt`.

Currently this package is available in these package repositories:

- **https://cppget.org/fmt/** for released and published versions.
- The git repository with the sources of the build2 package of fmt
  for unreleased or custom revisions of `fmt`.

**Usage:**

- `build2` package name: `fmt`
- Library target name : `lib{fmt}`

For example, to make your `build2` project depend on `fmt`:

- Add one of the repositories to your configurations, or in your
  `repositories.manifest`, if not already there:

  ```
  :
  role: prerequisite
  location: https://pkg.cppget.org/1/stable
  ```
- Add this package as a dependency to your `./manifest` file
  (example for `v7.0.x`):

  ```
  depends: fmt ~7.0.0
  ```
- Import the target and use it as a prerequisite to your own target
  using `fmt` in the appropriate `buildfile`:

  ```
  import fmt = fmt%lib{fmt}
  lib{mylib} : cxx{**} ... $fmt
  ```

Then build your project as usual with `b` or `bdep update`.

For `build2` newcomers or to get more details and use cases, you can read the
`build2`
toolchain introduction.

## Building the Documentation¶

To build the documentation you need the following software installed on your
system:

- Python with pip and virtualenv
- Doxygen
- Less with `less-plugin-clean-css`.
  Ubuntu doesn’t package the `clean-css` plugin so you should use `npm`
  instead of `apt` to install both `less` and the plugin:

  ```
  sudo npm install -g less less-plugin-clean-css.
  ```

First generate makefiles or project files using CMake as described in
the previous section. Then compile the `doc` target/project, for example:

```
make doc
```

This will generate the HTML documentation in `doc/html`.

## Conda¶

fmt can be installed on Linux, macOS and Windows with
Conda, using its
conda-forge
package, as follows:

```
conda install -c conda-forge fmt
```

## Vcpkg¶

You can download and install fmt using the vcpkg dependency manager:

```
git clone https://github.com/Microsoft/vcpkg.git
cd vcpkg
./bootstrap-vcpkg.sh
./vcpkg integrate install
./vcpkg install fmt
```

The fmt port in vcpkg is kept up to date by Microsoft team members and community
contributors. If the version is out of date, please create an issue or pull
request on the vcpkg repository.

## LHelper¶

You can download and install fmt using
lhelper dependency manager:

```
lhelper activate <some-environment>
lhelper install fmt
```

All the recipes for lhelper are kept in the
lhelper’s recipe repository.

## Android NDK¶

fmt provides Android.mk file that can be used to build the library
with Android NDK.
For an example of using fmt with Android NDK, see the
android-ndk-example
repository.

## Homebrew¶

fmt can be installed on OS X using Homebrew:

```
brew install fmt
```

© Copyright 2012-present, Victor Zverovich.
Created using Sphinx 3.3.0.
